# Supplementary material for: Loss of Zonula Occludens-1 (ZO-1) Enhances Angiogenic Signaling in Ovarian Cancer Cells
Source: Int J Mol Sci. 2025 Aug 29;26(17):8389. doi: 10.3390/ijms26178389 (PMC12429131; doi:10.3390/ijms26178389)
Supplement: Supplementary file 1 [file ijms-26-08389-s001.zip › ARRIVE guideline.pdf]

# ARRIVE 2.0 Checklist (Author Completed Example)

| Item | Content |
| --- | --- |
| 1. Study design | Two groups of mice (SKOV3 vs. ZO-1 KO SKOV3) were compared in Matrigel plug assays. Each group received subcutaneous injection of Matrigel-cell mixture. Endpoint: angiogenesis within Matrigel plugs after 14 days. |
| 2. Sample size | n = 5 mice per group (total 10). Number determined based on pilot experiments and similar published studies. No animals were excluded. |
| 3. Inclusion and exclusion criteria | All mice meeting age (6–8 weeks), sex (female), and strain (BALB/c nude) requirements were included. No exclusion criteria applied post hoc. |
| 4. Randomization | Animals were randomly assigned to experimental groups before injection. |
| 5. Blinding | Histological analysis of Matrigel plugs was performed by investigators blinded to group allocation. |
| 6. Outcome measures | Primary outcome: vascularization within Matrigel plugs, assessed by histology (H&E, immunostaining). |
| 7. Statistical methods | Data analyzed using GraphPad Prism. Comparisons performed using Student's t-test (two-tailed). $p < 0.05$ considered statistically significant. |
| 8. Experimental animals | Species: Mus musculus (mouse). Strain: BALB/c nude. Sex: female. Age: 6–8 weeks. Supplier: Hana Biotech (Korea). |
| 9. Experimental procedures | Cells ($1 \times 10^6$) mixed with 250 µL Matrigel + 50 µL medium (total 300 µL). Subcutaneous injection into both flanks. Housing: SPF facility, 12 h light/dark cycle, food and water ad libitum. Endpoint: 14 days after injection. Euthanasia: $CO_2$ inhalation under anesthesia, according to IACUC guidelines. |
| 10. Results | All animals completed the study (no loss). Results presented in Figures 3 of the manuscript. |

---

# ☐ Compliance

- All procedures complied with the Animal Protection Act of Korea, the Laboratory Animal Act of Korea, the ARRIVE guidelines (2.0), and the NIH Guide for the Care and Use of Laboratory Animals (8th edition).
- Protocol approval: Kosin University College of Medicine IACUC (Approval No. KUCMIACUC [KMAP-24-09]).
