# Supplementary material for: Loss of Zonula Occludens-1 (ZO-1) Enhances Angiogenic Signaling in Ovarian Cancer Cells
Source: Int J Mol Sci. 2025 Aug 29;26(17):8389. doi: 10.3390/ijms26178389 (PMC12429131; doi:10.3390/ijms26178389)
Supplement: Supplementary file 1 [file ijms-26-08389-s001.zip › ijms-3816063 (2).pdf]

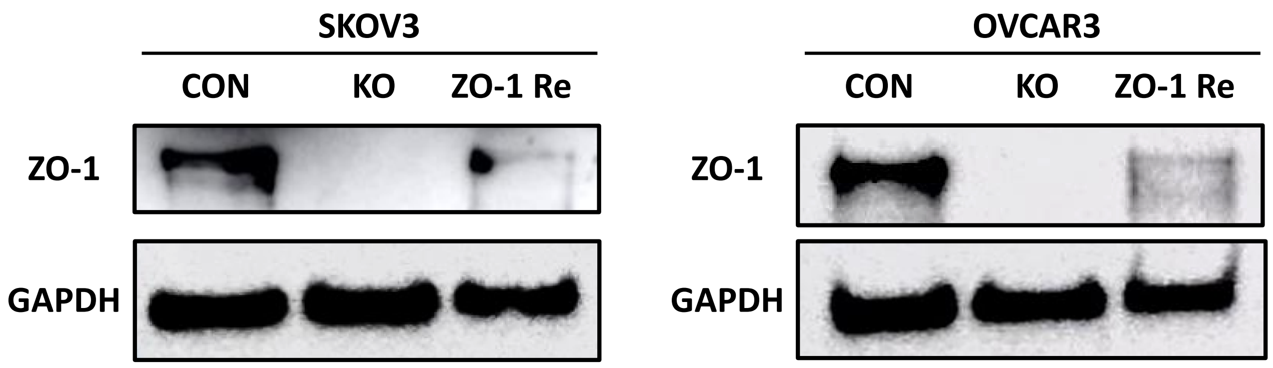

Supplemental Figure S1. Proteins were extracted from ZO-1-rescued cell lines and verified by Western blot analysis.

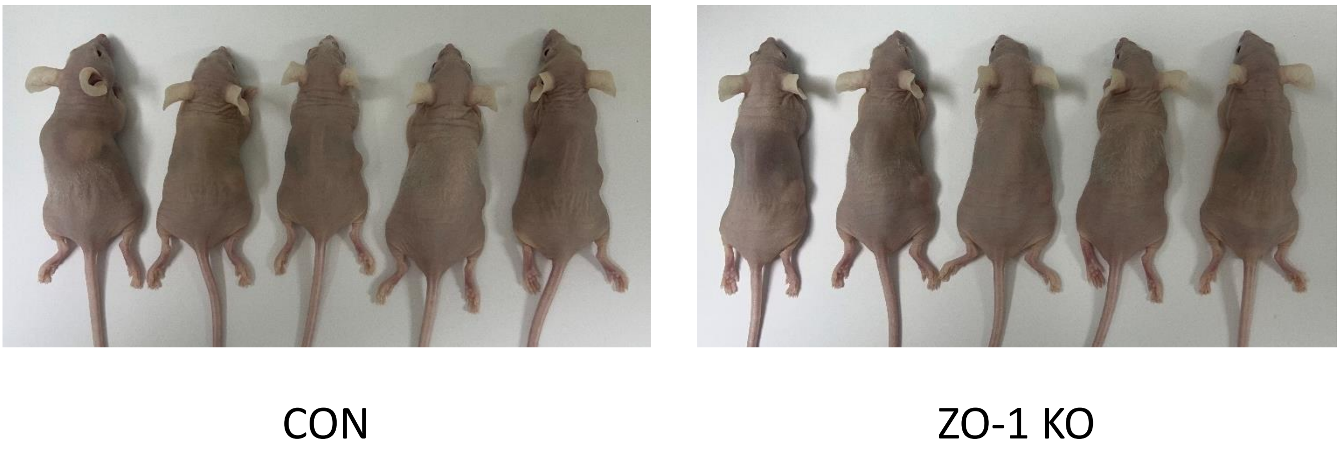

Supplemental Figure S2. To evaluate angiogenesis in vivo, control and ZO-1 KO SKOV3 cells were mixed with Matrigel and subcutaneously injected into BALB/c nude mice (n = 5 per group).
