# Supplementary material for: Loss of Zonula Occludens-1 (ZO-1) Enhances Angiogenic Signaling in Ovarian Cancer Cells
Source: Int J Mol Sci. 2025 Aug 29;26(17):8389. doi: 10.3390/ijms26178389 (PMC12429131; doi:10.3390/ijms26178389)
Supplement: Supplementary file 1 [file ijms-26-08389-s001.zip › ijms-3816063 (3).pdf]

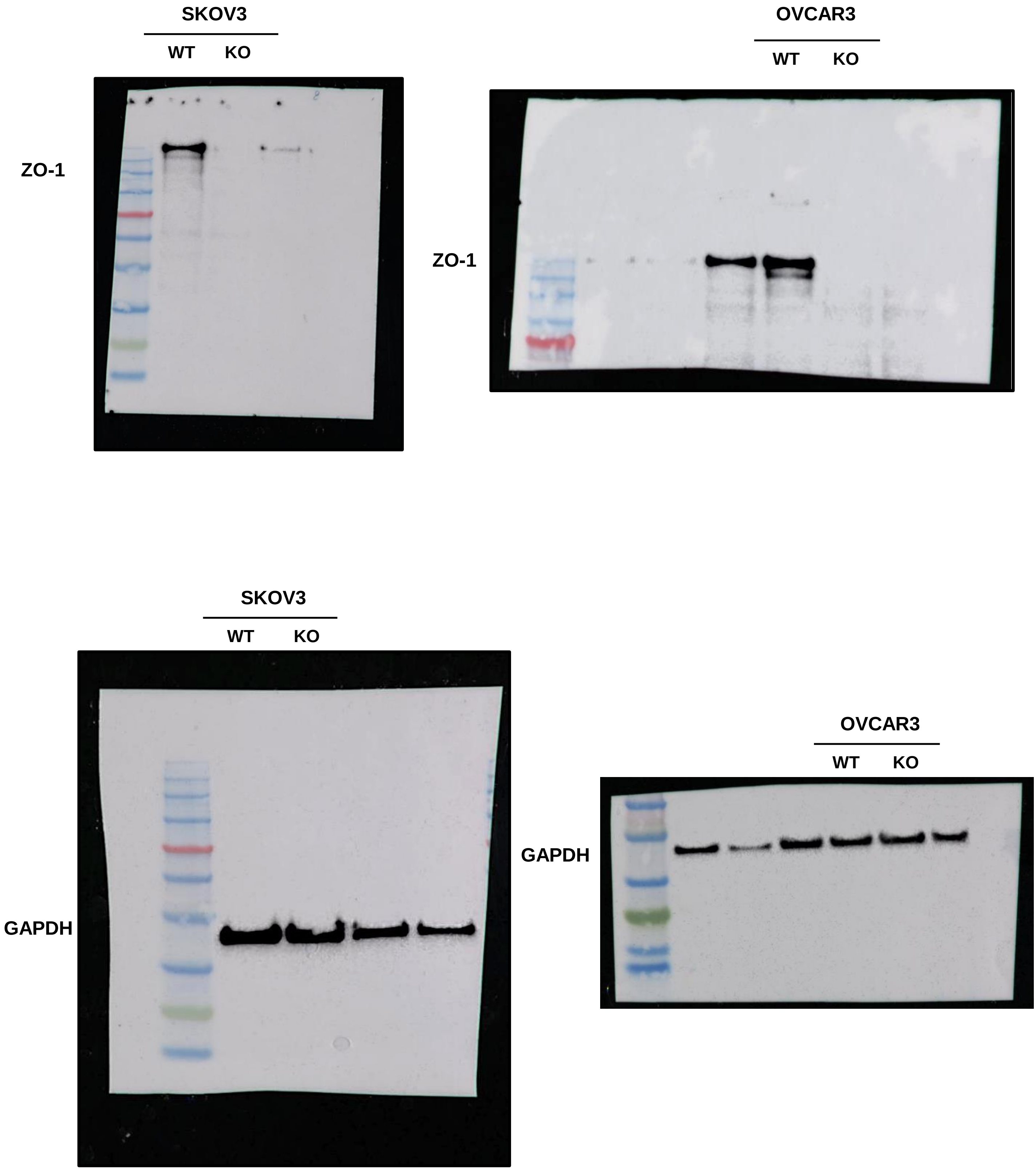

The original membrane image for panel C in Figure 1.

SKOV3

CON KO

IL8

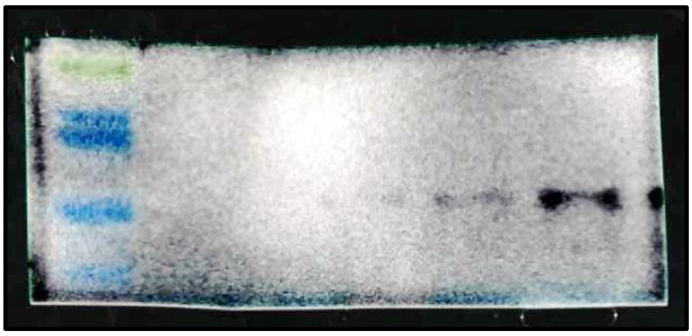

OVCAR3

CON KO

IL8

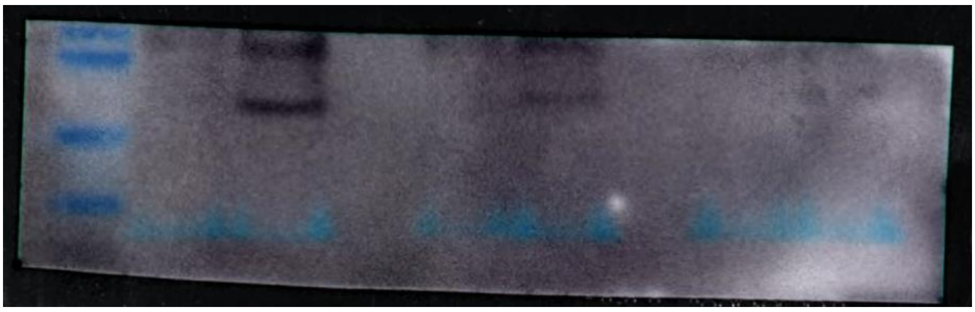

SKOV3

CON KO

GAPDH

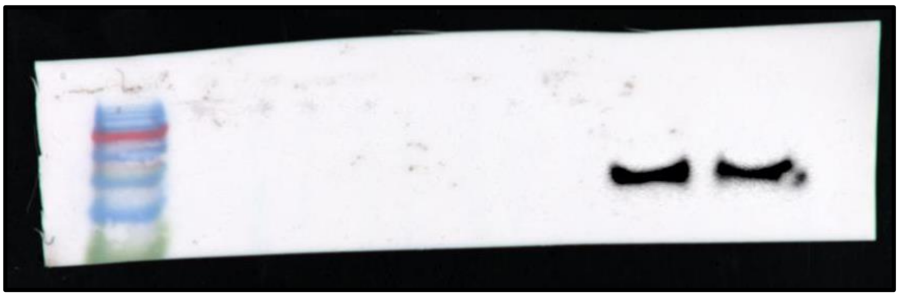

OVCAR3

CON KO

GAPDH

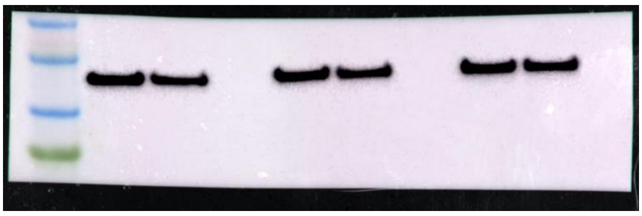

SKOV3 OVCAR3

CON KO CON KO

KLF5

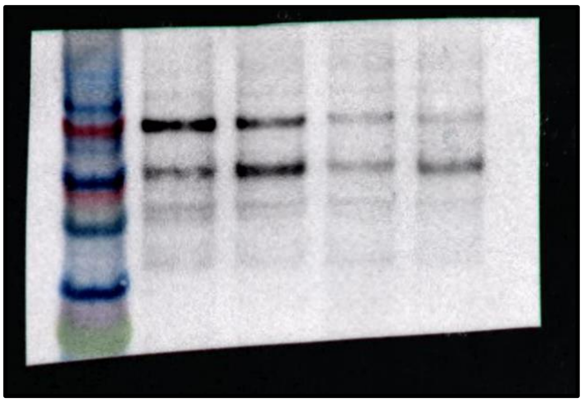

SKOV3 OVCAR3

CON KO CON KO

GAPDH

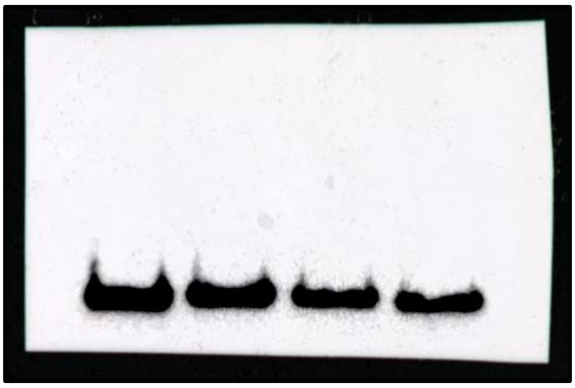

The original membrane image for panel D in Figure 4.

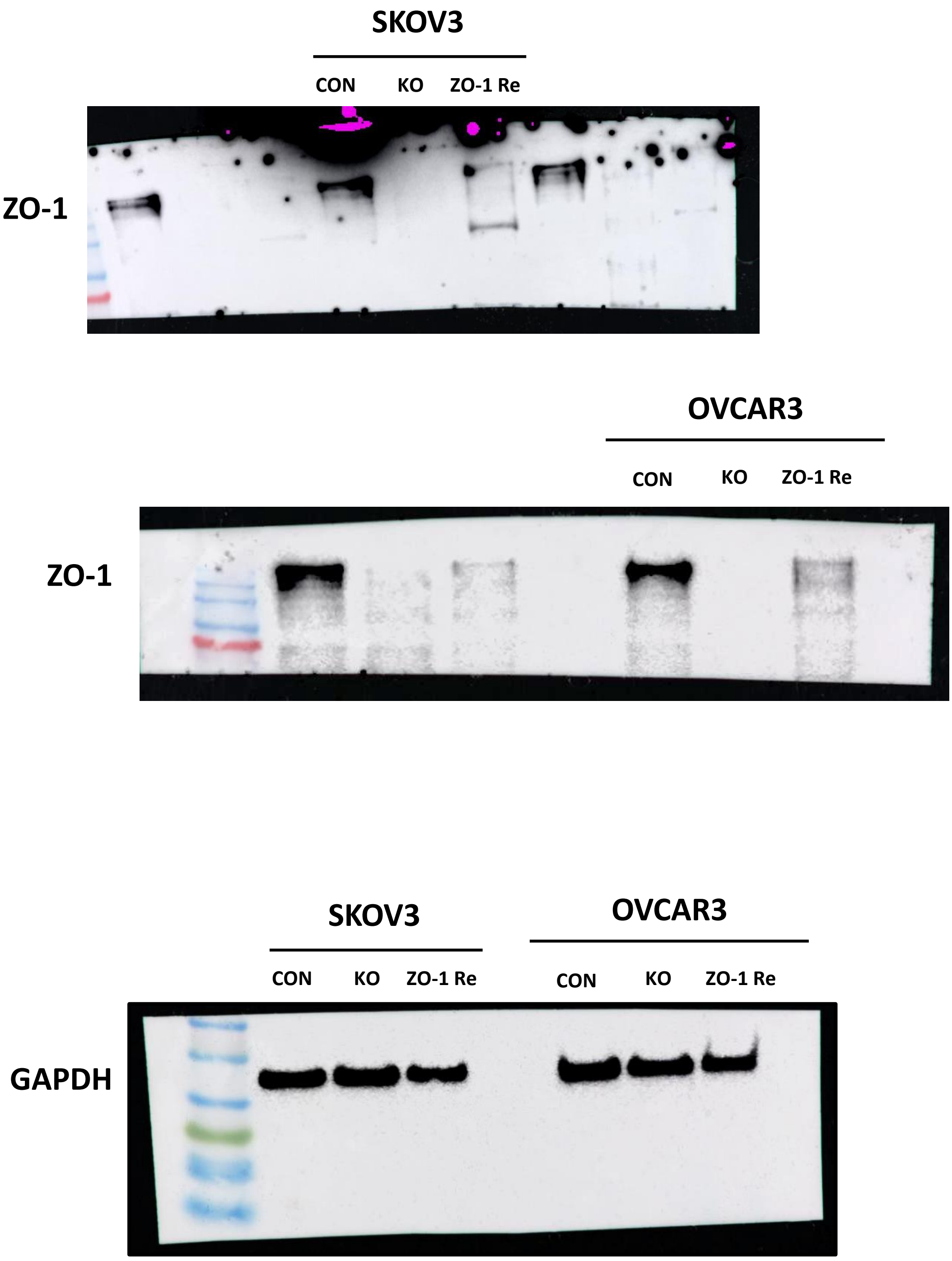

The original membrane image in Supplemental Figure S1
